# Microscopic Characterization of Individual Aerosol Particles in a Typical Industrial City and Its Surrounding Rural Areas in China

**DOI:** 10.3390/toxics12070525

**Published:** 2024-07-22

**Authors:** Yunfei Su, Yuhan Long, Xunzhe Yao, Chunying Chen, Wei Sun, Rui Zhao, Junke Zhang

**Affiliations:** School of Environmental Science and Engineering, Southwest Jiaotong University, Chengdu 611756, China; 1453978034@163.com (Y.S.);

**Keywords:** individual aerosol particles, morphology, mixing state, particle size distribution, regional transport

## Abstract

Transmission electron microscopy was used to analyze individual aerosol particles collected in Lanzhou (urban site) and its surrounding areas (rural site) in early 2023. The results revealed that from the pre-Spring Festival period to the Spring Festival period, the main pollutants at the urban site decreased significantly, while the PM_2.5_ and SO_2_ concentrations increased at the rural site. During the entire sampling period, the main particles at the urban site were organic matter (OM), secondary inorganic aerosols (SIA), and OM-SIA particles, while those at the rural site were OM, SIA, and soot particles. The degree of external mixing of single particles in both sites increased from the pre-Spring Festival period to the Spring Festival period. The proportion of the OM particles increased by 11% at the urban site, and the proportion of SIA particles increased by 24% at the rural site. During the Spring Festival, the aging of the soot particles was enhanced at the urban site and weakened at the rural site. At the urban site, the SIA particle size was more strongly correlated with the thickness of the OM coating during the pre-Spring Festival period, while the correlation was stronger at the rural site during the Spring Festival.

## 1. Introduction

Since the implementation of China’s reform and opening-up policy, the rapid urbanization and industrialization processes, coupled with the steady economic growth, have led to the emission of significant quantities of pollutants, resulting in frequent haze pollution across most regions of China [1]. Numerous studies have confirmed that high-intensity haze can have deleterious effects on human health, leading to premature deaths and cardiovascular and respiratory diseases [2,3]. Furthermore, haze can directly and indirectly alter the climate, influencing precipitation patterns and marine ecosystems [4,5,6]. Although the Chinese government has implemented a series of pollution reduction policies, including the Air Pollution Prevention and Control Action Plan (2013–2017) and the Three-Year Action Plan to Win the Blue Sky Defense War (2018–2020) [7,8,9], due to various factors, such as unfavorable meteorological conditions (including a low wind speed and high relative humidity), household heating, exhaust emissions from motor vehicles, industrial activities, and the long-distance transport of atmospheric pollutants, a considerable number of cities across China still experienced severe haze pollution incidents throughout the winter months in recent years [10,11,12]. In addition, the current scientific understanding of the sources, formation mechanisms, and impacts of haze pollution remains insufficient [13,14,15]. 

Aerosol particles are crucial in triggering haze events [16,17]. Multiple studies have reported that in recent years, with the reduction of pollutant emissions in China, the characteristics, sources, formation mechanisms, and impacts of aerosol particles have undergone dynamic changes [11,18,19]. For instance, Zhou, et al. [20] observed that the contribution of nitrates to the aerosol particles in Beijing significantly increased from 2011/2012 to 2017/2018. In contrast, the contributions of sulfates, organic matter (OM), and chlorides decreased. Zhang, et al. [13] found that from 2016 to 2020, OM and nitrate (NO_3_^−^) played an increasingly prominent role in the formation of severe winter pollution in Chengdu, while the contribution of sulfate (SO_4_^2−^) decreased year by year. 

Lanzhou, with its unique valley-basin terrain, which greatly hinders pollution dispersal, is one of the world’s most polluted industrial cities [21,22,23]. In addition, statistical data indicate that nearly 20% of the total population of Gansu Province resides in Lanzhou and its surrounding areas where approximately 25% of fossil fuels are consumed for industrial, residential, and vehicular activities (http://data.stats.gov.cn/index.htm, accessed on 12 December 2023). This contributes to the regional haze pollution in Lanzhou and its neighboring regions, making it one of the primary environmental issues in Northwestern China [21,24,25]. Although significant improvements in the air quality have been observed in Lanzhou over the past decade, its PM_2.5_ concentration (33 μg/m^3^ in 2022) remains significantly higher than the World Health Organization’s guideline value (5 μg/m^3^). Furthermore, the air quality frequently reaches moderate to severe pollution levels during the winter months. 

Transmission electron microscopy (TEM) is an effective approach for investigating aerosol particles. It enables comprehensive characterization of the morphology, chemical composition, particle size, and mixing state of particles [4,26,27,28], which are crucial information for understanding the sources, formation mechanisms, and aging processes of particles [28,29,30]. Additionally, insights into the mixing state and morphology contribute to the understanding of the optical and hygroscopic properties of particles [4,28]. Although extensive research has been conducted by numerous scholars utilizing various offline and online observation methods to investigate the chemical composition, sources, formation mechanisms, and seasonal characteristics of aerosol particles in Lanzhou [25,31,32] and fruitful results have been obtained, few studies have utilized TEM specifically for the analysis of aerosol particles in this region. Therefore, in this study, we specifically observed and analyzed individual aerosol particles in Lanzhou and its surrounding rural areas in January 2023 using TEM method. By comparing the characteristics of the individual aerosol particles in urban (U1) and rural (R1) sites during the pre-Spring Festival period, as well as in urban (U2) and rural sites (R2) during the Spring Festival period, we obtained results that can provide scientific information for understanding the characteristics and formation mechanisms of atmospheric pollution in different regions of Lanzhou. 

## 2. Materials and Methods

### 2.1. Sampling Sites and Data Collection

**Figure 1:**
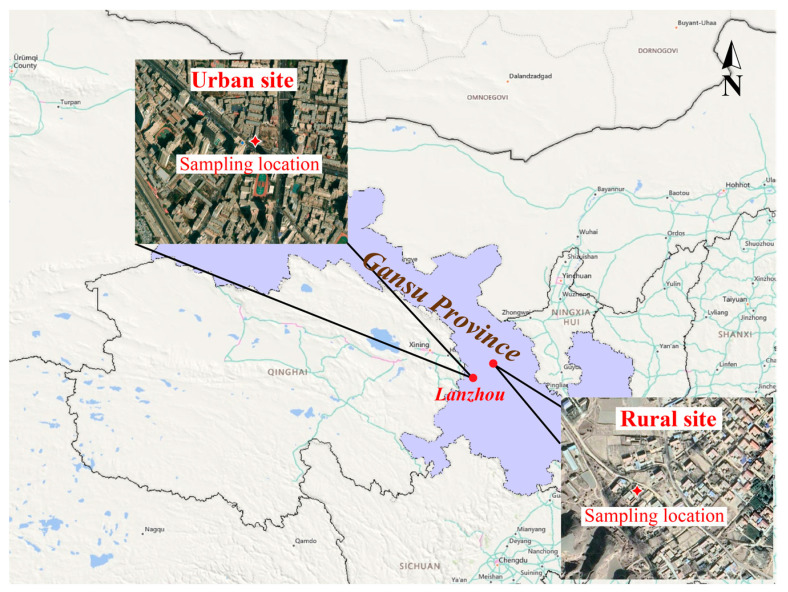
Sampling area in this study. The red stars represent the positions of the two sampling sites.

In this study, we utilized a DKL-2 sampler to collect individual aerosol particles in two distinct atmospheric environments in Lanzhou: urban and rural areas. The urban sampling site was located in the Chengguan District, Lanzhou City (36.03° N, 103.40° E), which is situated at the center of the city. The rural sampling site was located in Dalu Town, northeastern Lanzhou (36.33° N, 104.82° E) (Figure 1). The sampling at the urban site was conducted from 15–18 January 2023 and from 24–27 January 2023, representing the pre-Spring Festival period (U1) and the Spring Festival period (U2), respectively. Sampling at the rural site was conducted from 19–20 January 2023 and from 21–23 January 2023, representing the pre-Spring Festival period (R1) and the Spring Festival period (R2), respectively. The concentrations of air pollutants (e.g., PM_2.5_, SO_2_, NO_2_, CO, and O_3_) in both the urban and rural sites were obtained from the Ministry of Ecology and Environment of China (http://www.cnemc.cn/, accessed on 12 December 2023). In addition, the temperature (T) and relative humidity (RH) on each day were recorded simultaneously during the sampling.

### 2.2. Sampling and Analysis 

#### 2.2.1. Sample Collection

The sampling was conducted using a single-stage cascade impactor (DKL-2, Qingdao Genstar Electronic Technology Co., Ltd., Qingdao, China) equipped with a jet nozzle with a pore size of 0.5 mm in diameter, achieving a collection efficiency of 50% for aerosol particles with an aerodynamic diameter of 0.1 mm and a density of 2 g/cm^3^ [33,34]. The sampling flow rate was controlled at 1.0 L/min. The sampling duration ranged from 200 to 400 s, depending on the level of atmospheric pollution during the sampling, to ensure that the collected single particles were dispersed and did not overlap. The sampling membrane consisted of copper mesh coated with a carbon film (carbon type-B, 300-mesh copper; Tianld Co., Kaifeng, China). The single-stage cascade impactor installed on the single particle sampler is composed of two parts twisted together, with a flat surface for placing a copper mesh between them. The copper mesh is placed after the cascade impactor and connected to the inlet of the single particle sampler, so particles will be captured by the copper mesh. After the collection, the sampling membrane was placed in a sealed, dry plastic capsule and stored in a constant T (25 °C) and RH (20 ± 3)% container for subsequent analysis. 

#### 2.2.2. Individual Particle Measurements and Analysis

Due to the inhomogeneous distribution of the single-particle samples on the copper mesh, with larger particles clustered near the center and smaller particles dispersed toward the edges, it was necessary to ensure representative results for the particle analysis. Therefore, 4–5 grids were randomly selected from the center to the edge of the sample membrane for observation and analysis. Particle analysis was conducted on the selected areas of the sampling membrane using a TEM with a 200 kV accelerating voltage (JEM-2100, JEOL Ltd., Tokyo, Japan) and equipped with an energy-dispersive X-ray spectrometer (EDS, INCA X-MaxN 80T, Oxford Instruments, Oxford, UK). Subsequently, the TEM images were manually processed to determine the particle area, perimeter, and equivalent circular diameter (ECDs), and to determine the particle morphology and mixing state. The particles were then analyzed by EDS to conduct semi-quantitative analysis of the elements with atomic weights corresponding to C and above. Since the sampling membrane was made of copper, Cu was excluded from the analysis [17,35]. The EDS measurement time was set at 15 s. After labor-intensive operations, the samples were finally statistically analyzed to obtain the particle types and size distributions corresponding to the two sites. Finally, based on 26 samples collected at two sites, a total of 7268 and 8084 single-particle data were obtained for the urban site and rural site, respectively. 

#### 2.2.3. Regional Transport Analysis

The hybrid single-particle Lagrangian integrated trajectory (HYSPLIT) model is one of the most widely used atmospheric transport models for simulating the atmospheric transport, dispersion, and deposition of pollutants and hazardous substances [16]. In this study, the HYSPLIT-4 model was utilized to calculate the 48-h backward trajectories of the air masses that arrived at the urban and rural sites during the sampling periods (http://ready.arl.noaa.gov/HYSPLIT.php, accessed on 12 December 2023). The model was run with an hourly resolution and a height setting of 300 m.

Concentration weighted trajectory (CWT) analysis was conducted to calculate the trajectory weighted concentration. In the CWT method, each grid cell is assigned by averaging the weighted concentration of the sample pollutants that have correlated tracks across the grid cells:

$$C_{\mathrm{ij}}=\frac{\sum_{l=1}^{M}c_{l}\tau_{\mathrm{ijl}}}{\sum_{l=1}^{M}\tau_{\mathrm{ijl}}},$$

where *C_ij_* is the average weighted concentration of the *ij*th cell, *l* is the trajectory index, *M* is the total number of trajectories, *C_l_* is the pollutant concentration, and *τ_ijl_* is the residence time of *l*.

The CWT grids covered domains in the range of 30–46° N and 85–108° E at a resolution of 0.6° × 0.6°. To mitigate the impact of small values of *n_ij_*, the CWT values were multiplied by an arbitrary weight function *W_ij_* to better reflect the uncertainty of the values in these cells. The *W_ij_* values used for the current analysis were as follows:

$$W_{\mathrm{ij}}=\left\{\begin{array}{cc} 1.00 & (n_{\mathrm{ij}}\geq 3n_{\mathrm{ave}}),\\ 0.70 & (3n_{\mathrm{ave}}>n_{\mathrm{ij}}\geq 1.5n_{\mathrm{ave}}),\\ 0.42 & (1.5n_{\mathrm{ave}}>n_{\mathrm{ij}}\geq n_{\mathrm{ave}}),\\ 0.17 & (n_{\mathrm{ave}}>n_{\mathrm{ij}}), \end{array}\right.$$

where *n_ave_* is the average number of trajectory endpoints per grid cell. The weighted CWT (WCWT) was calculated as follows:

WCWT*_ij_* = *W_ij_* × *C_ij_.*

## 3. Results and Discussion

### 3.1. Overview of Meteorological and Pollution Characteristics

The average temperature and relative humidity at the urban site during the entire observation period were −9.5 ± 4.6 °C and 39.1 ± 14.7%, respectively, which were lower than those at the rural site during the same period (−9.2 ± 3.9 °C and 62.0 ± 26.9%, respectively). Previous studies have shown that high temperatures can cause the formation of secondary components such as sulfates and secondary organic compounds in PM_2.5_, but they also lead to the decomposition of ammonium nitrate [36,37,38]. At the same time, high relative humidity can promote the formation of secondary pollutants in the liquid phase, causing their contribution to increase [39,40]. It can be seen that temperature and relative humidity have a direct and decisive effect on the chemical composition of particulate matter. Therefore, the differences in meteorological conditions between rural and urban areas may determine the differences in pollution mechanisms between the two sites. Additionally, the average wind speed at the urban site (6.3 ± 3.5 m/s) was lower than that at the rural site (7.5 ± 5.6 m/s), suggesting the occurrence of unfavorable meteorological conditions for the dispersion of pollutants in urban environments. Specifically, during period U1, the average temperature and wind speed were the lowest (−11.9 ± 4.9 °C and 4.3 ± 2.9 m/s, respectively), indicating that the horizontal dispersion of the pollutants was weaker during U1 than during the other periods. In contrast, the average temperature was the highest during R1 (−7.5 ± 0.7 °C), while the highest relative humidity (78.3 ± 20.2%) occurred during R2. 

**Figure 2:**
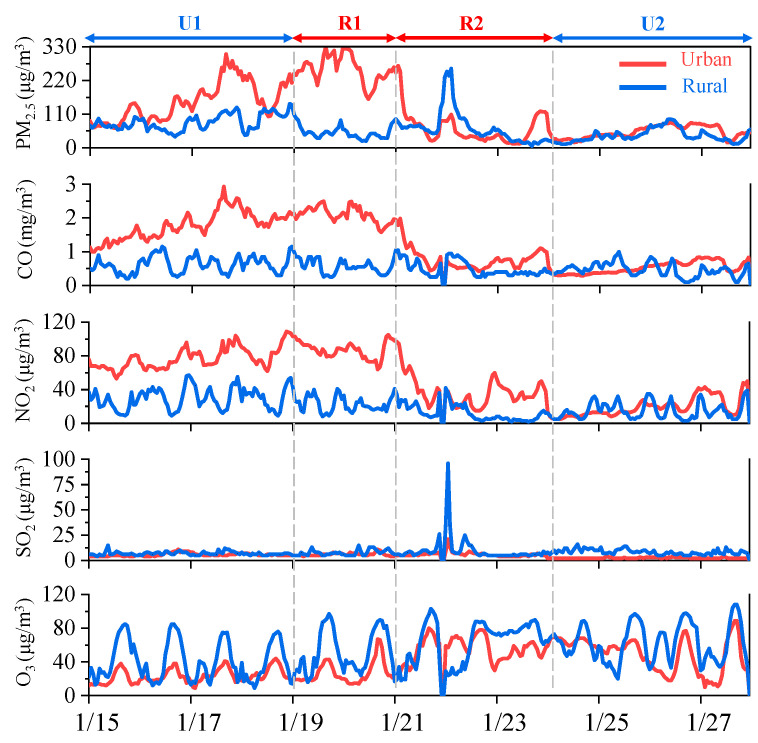
Temporal variation in the PM_2.5_, SO_2_, NO_2_, and O_3_ mass concentrations during the sampling periods.

As important precursors of particulate matter, gaseous pollutants are also important parameters for evaluating air quality. As shown in Figure 2, the SO_2_ concentration at the urban site (4.0 ± 2.0 µg/m^3^) was significantly lower than the Chinese National Ambient Air Quality Standard (CNAAQS, 60 µg/m^3^); it was only 41.2% of the concentration at the rural site (9.7 ± 9.5 µg/m^3^). NO_2_ was significantly influenced by vehicle exhaust emissions [17], and its concentration at the urban site (48.1 ± 31.4 µg/m^3^) was approximately 3.3 times that at the rural site (14.4 ± 9.6 µg/m^3^). This disparity highlights the impact of urban vehicular emissions on local NO_2_ concentrations. The CO concentration at the urban site (1.2 ± 0.7 µg/m^3^) was approximately 2.4 times higher than that at the rural site (0.5 ± 0.2 µg/m^3^). Furthermore, there was a significant correlation between the CO and NO_2_ collected at the two sampling sites, indicating their common origin from motor vehicle emissions. Conversely, as a secondary pollutant generated by NO_X_ and VOCs under sunlight, the rural site had a higher O_3_ concentration (61.4 ± 23.0 µg/m^3^), approximately 1.7 times higher than that at the urban site (36.4 ± 20.3 µg/m^3^). The rural site had a higher O_3_ concentration (61.4 ± 23.0 µg/m^3^), approximately 1.7 times higher than that at the urban site (36.4 ± 20.3 µg/m^3^). The average mass concentrations of PM_2.5_ at the urban and rural sites were 100.7 ± 67.5 µg/m^3^ and 50.7 ± 42.4 µg/m^3^, respectively, which were 2.9 and 1.5 times the CNAAQS (35 μg/m^3^), respectively, and 20.1 and 10.1 times the World Health Organization (WHO) (an annual average of 5 μg/m^3^) guidelines, respectively. 

There were significant differences in the variations in the gaseous pollutants during the different periods. Specifically, from U1 to U2, the average concentrations of SO_2_, CO, and NO_2_ exhibited a decreasing trend, with values decreasing from 5.6 ± 1.5 µg/m^3^, 1.8 ± 0.4 mg/m^3^, and 76.9 ± 31.4 µg/m^3^ to 2.3 ± 0.5 µg/m^3^, 0.5 ± 0.2 mg/m^3^, and 19.1 ± 11.5 µg/m^3^, respectively, i.e., decreases of 58.9%, 72.2%, and 75.2%, respectively. This was related to the weakening of motor vehicle activities and the temporary cessation of numerous outdoor operations and urban construction, as well as the shutdown of industrial enterprises during the Spring Festival. Consistent with the varying trends of these crucial gaseous precursors, the average concentration of PM_2.5_ decreased notably, from 149.6 ± 62.2 µg/m^3^ to 51.3 ± 20.0 µg/m^3^, i.e., a decrease of 65.7%. In contrast, the concentration of O_3_ increased from 23.3 ± 8.5 µg/m^3^ (U1) to 49.7 ± 20.1 µg/m^3^ (U2), exhibiting a different pattern compared to the other pollutants that exhibited significant decreases. 

From R1 to R2, the average concentration of SO_2_ increased from 8.6 ± 3.8 µg/m^3^ to 10.4 ± 11.8 µg/m^3^, i.e., an increase of 20.9%. Conversely, the average concentration of NO_2_ decreased from 19.8 ± 8.1 µg/m^3^ to 10.9 ± 8.8 µg/m^3^. The average concentration of PM_2.5_ decreased slightly from 54.7 ± 21.5 µg/m^3^ to 48.1 ± 51.5 µg/m^3^. The CO concentration was highly stable during both periods and remained constant at 0.5 ± 0.2 mg/m^3^. Similar to the urban site, the concentration of O_3_ increased from 54.5 ± 26.7 µg/m^3^ to 65.8 ± 19.2 µg/m^3^. 

### 3.2. Classification and Mixing States of the Types of Particles

**Figure 3:**
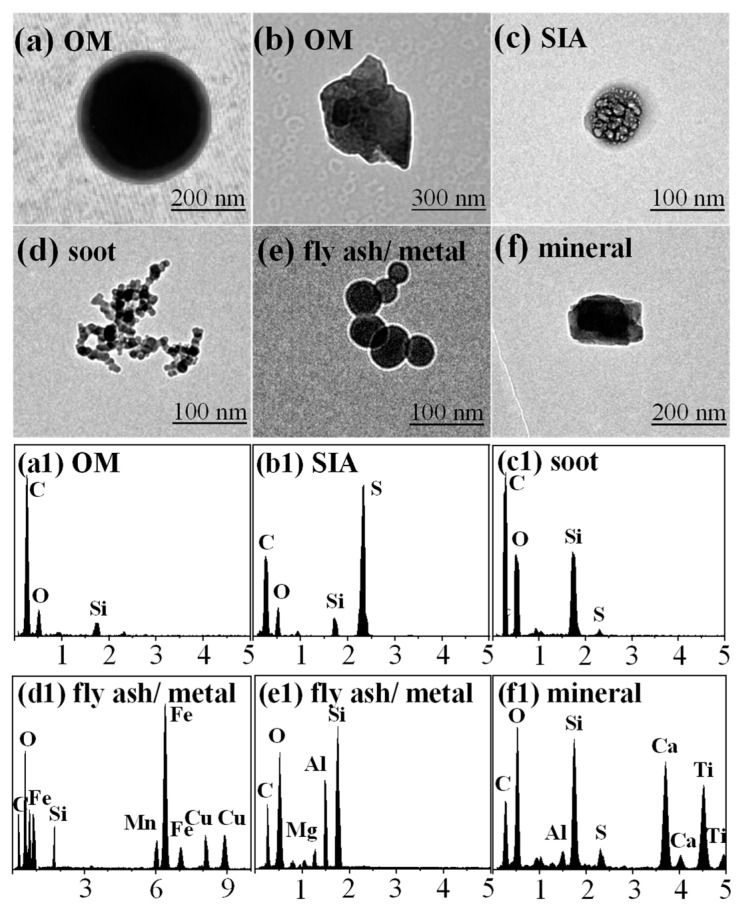
TEM images and EDS spectra of aerosol particles.

According to the morphologies and elemental compositions of particles, and referring to previous studies [17,28,41,42], particles that appeared individually without mixing with other particles were defined as externally mixed particles, while particles that were composed of two or more particles mixed were referred to as internally mixed particles. In this study, the externally mixed particle was divided into five categories: OM, secondary inorganic aerosols (SIA), soot, fly ash/metals, and minerals. The TEM image and EDS spectrum of each type of particle are shown in Figure 3. 

**Figure 4:**
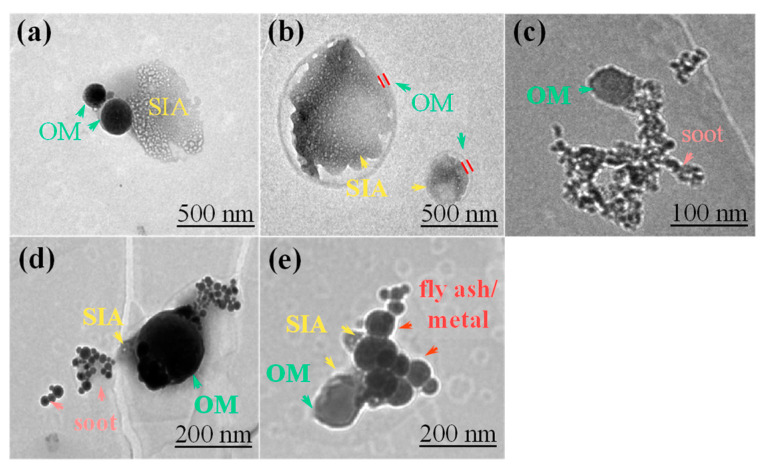
TEM images of internally mixed particles: (**a**) mixture of OM and SIA; (**b**) SIA coated with OM; (**c**) mixture of OM and soot; (**d**) mixture of OM, SIA, and soot; and (**e**) mixture of OM, SIA, and fly ash/metal particles.

The OM particles exhibited diverse morphologies and were primarily composed of C and O. These particles maintained a relatively stable morphology even under intense electron beam irradiation, which can be further classified into spherical, irregular, and coated shapes (Figure 3a,b and Figure 4b). The spherical and irregular OM particles were primarily emitted directly from coal or biomass burning [29,42], while the OM coatings were typically considered to be secondary organic matter produced via chemical oxidation of volatile organic compounds (VOCs) [17]. The SIA particles were primarily composed of C, O, Si, and S (Figure 3(b1)) and were produced via oxidation of SO_2_, NO_X_, and NH_3_, and they were often composed of mixtures of (NH_4_)_2_SO_4_ and NH_4_NO_3_ [28,29]. However, due to the reversible phase equilibrium with HNO_3_ and NH_3_, NH_4_NO_3_ aerosols were unstable under high temperature, high vacuum, and low humidity conditions, and were usually undetectable by TEM [28,43]. The soot particles, also known as black carbon (BC) or elemental carbon (EC), primarily consisted of aggregated spheres with diameters of 10–150 nm, and they often exhibited in chain-like or clustered forms (Figure 3d). The soot particles were mainly composed of C, as well as minor amounts of O and Si (Figure 3(c1)). They were generated from incomplete combustion processes of biomass and fossil fuels [44]. The fly ash/metal particles exhibited spherical morphologies (Figure 3e) and were primarily composed of C, O, Si, and metals such as Al, Fe, and Mn (Figure 3(d1,e1)). The sizes of these particles were mainly distributed in the ultrafine particle size range (<100 nm) and were typically emitted from coal-fired power plants, heavy industries, and refinery operations [28]. The mineral particles exhibited irregular shapes (Figure 3f) and were often generated from roads, deserts, and construction activities [29]. They were primarily composed of C, O, Al, Si, Ca, and Ti (Figure 3(f1)), and their sizes were mainly distributed in the coarse particle size range (>1 µm). 

Previous studies have demonstrated that—due to the large amount of VOCs in the atmosphere that adhere to the surface of primary and secondary substances through adsorption, coating, condensation, and gas-particle transformation processes to generate secondary organic compounds—the individual particles that originate from diverse sources undergo varying degrees of internal mixing with OM particles in the atmosphere, ultimately forming internal mixed particles [17,45,46,47]. Consequently, in this study, based on their types and mixing states, we classified the internally mixed particles into the following groups: OM-SIA, OM-soot, OM-SIA-soot, and OM-SIA-fly ash/metal (Figure 4). 

### 3.3. Individual Particle Characteristics at the Urban and Rural Sites

**Figure 5:**
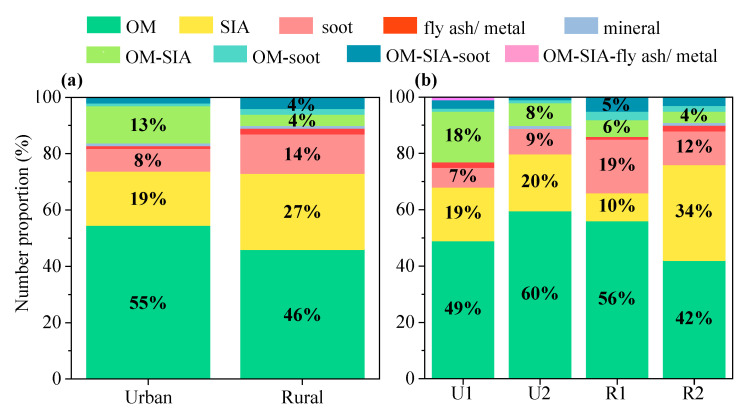
Compositions of the types of particles at the (**a**) urban and rural sites, and (**b**) at the two sampling sites during different periods.

As the provincial capital and background region of Gansu Province, Lanzhou City and Dalu Town exhibited differences in the composition and mixing of individual particles. To some extent, these differences reflected the different sources and atmospheric aging and mixing processes of the particles in these two distinct types of areas. These factors are important information for analyzing the formation of the pollution in each region. Figure 5a shows the compositions of the atmospheric individual particles at the two sampling sites during the entire observation period.

Regarding the relative proportions of the internally and externally mixed particles, the proportion of externally mixed particles was lower at the urban site (84%) than at the rural site (90%). Correspondingly, the proportion of internally mixed particles was higher at the urban site than at the rural site. In terms of the externally mixed particles, the proportion of OM particles was higher at the urban site (55%) than at the rural site (46%). However, the rural site had higher proportions of SIA and soot particles. Regarding the internally mixed particles, the proportion of OM-SIA particles at the rural site was 9% lower than that at the urban site. In contrast, the proportions of OM-soot and OM-SIA-soot particles were slightly higher (1% and 2%, respectively) at the rural site than at the urban site (Figure 5a). 

The compositions of the individual particles during the different periods were also significantly different (Figure 5b). First, the proportions of the externally mixed particles increased significantly from U1 to U2, with corresponding proportions of 77% and 90%, respectively. Second, in terms of the relative proportions of the various particle types, although the externally mixed particles were the main contributors during both periods, the proportion of the internally mixed OM-SIA particles in U1 (18%) was also significant. Further analysis of the other internally mixed particles revealed that the proportions of all of the internally mixed particles decreased to varying degrees during U2, reflecting the significant differences in the aging and mixing processes of the particles between these two periods. From R1 to R2, the externally mixed OM and soot particles decreased by 14% and 7%, respectively. Conversely, the proportion of the externally mixed SIA particles increased by 24%. Regarding the internally mixed particles, the proportions of the OM-SIA and OM-soot particles decreased by 2% and 1%, respectively. In addition, the overall proportion of the internally mixed particles was the lowest during R2 (9%) compared to the other three periods. Therefore, not only did the proportion of OM-related mixed particles decrease, but the internal mixing of carbonaceous particles with SIA weakened during R2. 

**Figure 6:**
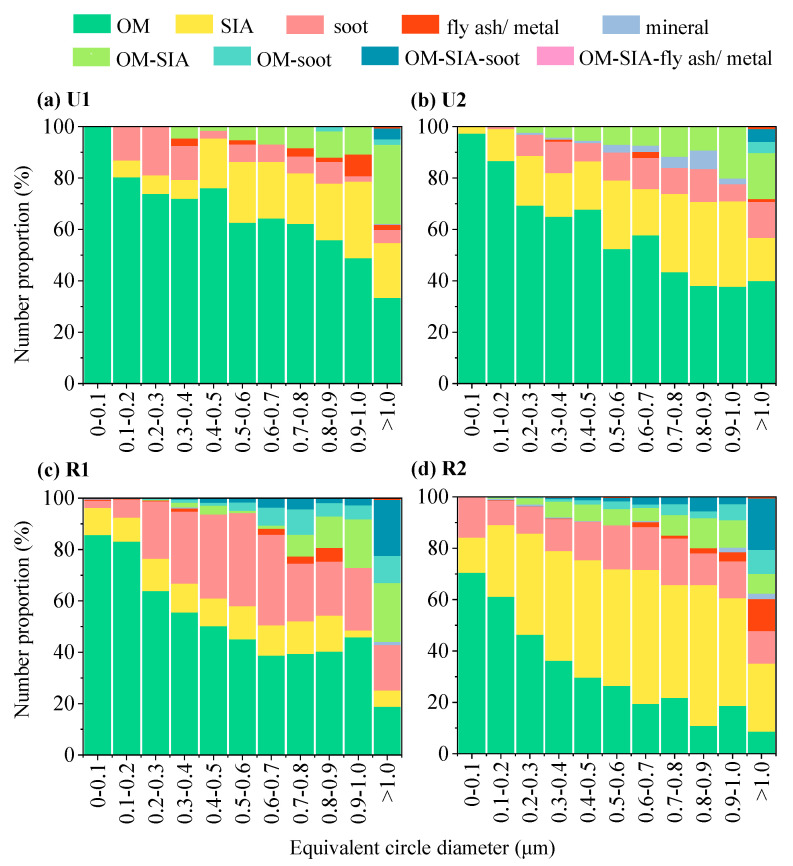
Proportions of the different types of particles by size (0.1 μm resolution) during the different periods.

Figure 6 shows the variations in the proportions of the different types of particulate matter with particle size in the urban and rural areas during the different periods. It can be seen that the particle size distributions of the OM particles were similar during U1 and U2. The highest peak occurred within the particle size range of 0.0–0.1 μm, and the proportions of OM reached 99.2% and 97.5% during U1 and U2, respectively. As the particle size increased, the proportion of the OM particles gradually decreased, and the lowest proportion occurred within the size ranges of >1.0 μm (33.5%) and 0.9–1.0 μm (37.8%) during U1 and U2, respectively. The distribution of the proportion of the SIA particles differed significantly during these two periods. During U1, the SIA particles were mainly distributed within the size range of >0.4 μm, accounting for approximately 19.4–29.8%. In contrast, during U2, the proportion of SIA particles rapidly increased with increasing particle size within the <1.0 μm range, with a maximum value of 32.9%. The soot particles were mainly distributed within the size range of 0.1–0.4 μm during U1, and the proportion of the soot particles was 13.0–18.8%. However, during U2, the proportion of the soot particles varied within the size range of >0.2 μm. In particular, the proportion of soot particles was significantly higher (12.1%) within the coarse particle size range (>0.6 μm) compared to that during U1 (6.8%).

At the rural site, when the particle size was less than 0.6 μm, the proportions of the OM particles decreased with increasing particle size in both periods. However, for particle sizes > 0.6 μm, the proportion of the OM particles remained stable during R1, and it even increased slightly (except for particle sizes > 1 μm), while the proportion of the OM particles decreased During R2. During R1, for particle sizes < 0.9 μm, the proportion of the SIA particles remained relatively stable (9.3–10.7%). However, during R2, for the particle size range of 0.0–0.9 μm, the proportion of the SIA particles generally increased, and the maximum value (54.8%) occurred within the 0.8–0.9 μm particle size range. The proportion of the soot particles initially increased and then decreased during R1, and the highest value (35.3%) occurred within the particle size range of 0.5–0.6 μm. However, during R2, the proportion of the soot particles did not exhibit a clear change trend, and it varied between 9.5% and 18.2%. The OM-SIA particles were mainly distributed in the coarse particle size range during R1, and their proportion rapidly increased for particle sizes > 0.7 μm. However, during R2, the OM-SIA particles were distributed within the particle size range of 0.2–1.0 μm, and their proportion slightly increased for particle sizes > 0.2 μm. The proportions of OM-SIA-soot particles were generally consistent during R1 and R2. 

### 3.4. Regional Transport Analysis

#### 3.4.1. Backward Trajectory Analysis

**Figure 7:**
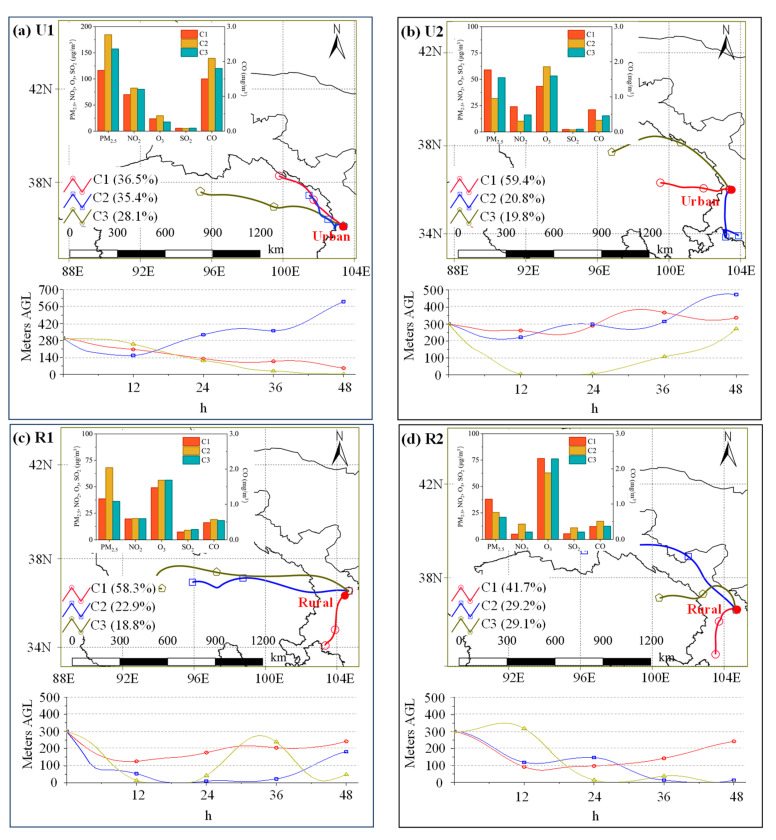
The 48-h backward trajectories of the air masses that arrived at the two sampling sites during the four periods and the pollutant concentrations of the three clusters. The numbers in parentheses are the proportions of the trajectories.

Figure 7 shows the air masses that affected both the urban and rural sites during the pre-Spring Festival and Spring Festival periods. The air masses were classified into three clusters (C1, C2, and C3). During U1, the proportions of these three types of air masses were close (28.1–36.5%). C1 originated in the northeastern part of Qinghai Province, accounted for 36.5% of all trajectories, and arrived in Lanzhou at a height of 300 m. It had a relatively fast speed starting in the middle of the Qilian Mountains. C2 had the highest height and slowest speed, accounted for 35.4% of the total trajectories and originated in the northeastern part of Qinghai Province. C3 accounted for 28.1% of the total trajectories, was a near-surface transport air mass, originated at the intersection of the Altun Mountains and the Qilian Mountains, and passed through significant industrial cities in Qinghai such as Golmud and Delingha. The PM_2.5_, NO_2_, and CO concentrations of C1 were lower than those of C2 and C3. Conversely, C3 had relatively high concentrations of most pollutants, which were second only to those of C2. The differences in the PM_2.5_ concentrations of the various air masses were highly consistent with the results of the potential source analysis, indicating that the regions with high pollutant concentrations corresponded to the areas with strong potential sources and vice versa (see Section 3.4.2).

In contrast to U1, there were significant differences in the proportions of the three air masses during U2 (Figure 7b). C1 accounted for the highest proportion of all of the trajectories (59.4%). Yet, it had transport heights of less than 400 m. C2 accounted for 20.8% of all trajectories and had transport heights of greater than 200 m. C3 had the longest trajectory and the highest speed, and its transport height was less than those of C2 and C1. The PM_2.5_, NO_2_, SO_2_, and CO concentrations were the highest in C1, followed by those in C3. However, the O_3_ concentration of C2 was the highest among the three air masses. 

During R1, C1, C2, and C3 accounted for 58.3%, 22.9%, and 18.8% of all of the trajectories, respectively, and it originated from southern Gansu Province and western Qinghai Province (Figure 7c). Except for the PM_2.5_ concentration, the concentrations of the pollutants associated with these air masses were quite similar. C2 had high concentrations of most of the pollutants. During R2, C1 (41.7%) originated from the southern part of Gansu Province, specifically from the Gannan Tibetan Autonomous Prefecture. C2 accounted for 29.2%, originated from the western part of Gansu, and traveled at the highest speed from Jiuquan to the rural sampling site via Jiayuguan and Wuwei cities. The contribution of C3 was similar to that of C2, accounting for 29.1%, and it originated from the Qinghai Lake. It reached the rural sampling site via Hainan Tibetan Autonomous Prefecture, Xining, and Haidong and traveled at a high speed. The NO_2_, SO_2_, and CO concentrations of C2 were the highest, followed by those of C3. However, C1 had the highest PM_2.5_ and O_3_ concentrations. 

#### 3.4.2. WCWT Analysis Results

**Figure 8:**
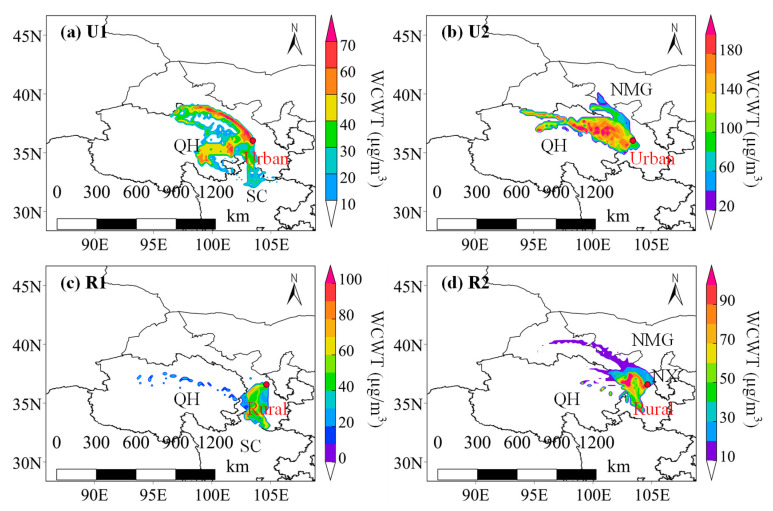
WCWT maps of the PM_2.5_ concentration during (**a**) U1, (**b**) U2, (**c**) R1, and (**d**) R2 (QH: Qinghai Province; NMG: Inner Mongolia Autonomous Region; SC: Sichuan Province; NX: Ningxia Hui Autonomous Region).

To further investigate the regional differences in the transport characteristics of the pollutants at the urban and rural sites, a CWT model was employed to analyze the potential source regions of PM_2.5_ (Figure 8). From U1 to U2, despite the significant reduction in the contribution from the boundary lines between the two provinces and the disappearance of the contribution from the central part of Qinghai Province, a very strong PM_2.5_ contribution area emerged in the northeastern part of Qinghai Province. The PM_2.5_ concentration contributed by a large part of the area exceeded 180 µg/m^3^, which was more than twice the highest contribution during U1 (70 µg/m^3^). During U2, the majority of the areas made potential PM_2.5_ concentration contributions of greater than 120 µg/m^3^, reflecting the significance of long-distance regional transport to urban pollution, especially during the Spring Festival.

As can be seen from Figure 8c,d, the rural site was significantly influenced by the transport of PM_2.5_ from the urban site. During R1, the high WCWT areas for the PM_2.5_ concentration were primarily located in the urban area in Lanzhou and southern Gansu, and the maximum concentration contribution was less than 80 µg/m^3^. From R1 to R2, the potential source area in southern Gansu decreased, while the source area and concentration contribution in the urban area in Lanzhou significantly increased, and the maximum contribution exceeded 90 µg/m^3^. At this time, the potential source area in central Gansu extended toward western Gansu, encompassing parts of southern Inner Mongolia and western Ningxia. During R2, the contribution from Lanzhou was significantly higher than that from the other regions, reflecting the prominent impact of Lanzhou on the pollution in its surrounding rural areas.

### 3.5. Comparison of Soot Particles at the Urban and Rural Sites

**Figure 9:**
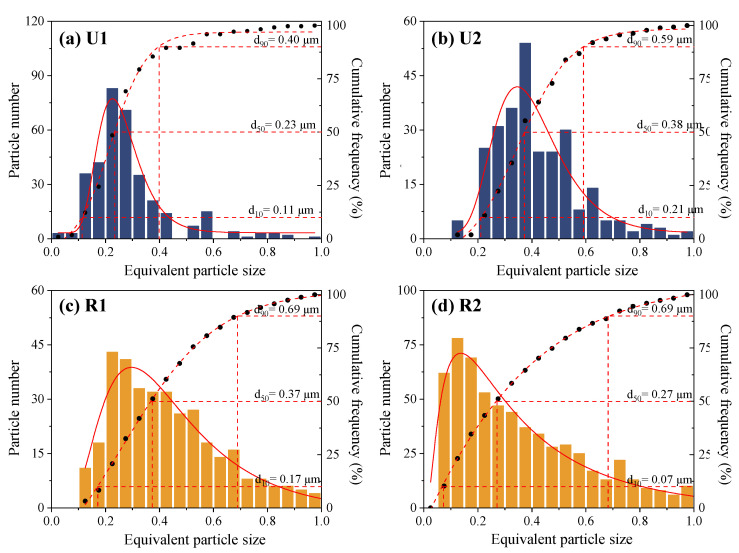
Size distributions of soot particles collected during (**a**) U1, (**b**) U2, (**c**) R1, and (**d**) R2. The size distribution with a single peak was fitted using a lognormal distribution.

Soot particles can directly absorb solar radiation, subsequently affecting the climate, cloud formation, and surface reflectance through deposition of the soot particles on ice and snow [44,48]. As a result, the contribution of the soot particles was second only to that of carbon dioxide in terms of anthropogenic radiative forcing [49,50]. In addition, the soot particles significantly enhanced the oxidative capacity of the atmosphere, promoting the formation of secondary aerosols [51,52]. Furthermore, soot particles can have a significant impact on human health as they contain polycyclic aromatic hydrocarbons (PAHs), and their mixture with secondary aerosols can increase the toxicity of the particles [15]. As a consequence of the atmospheric aging process, the chemical composition and microstructure of soot particles will change, further intensifying their impacts on human health [50,53]. In this study, the number contribution of the soot particles is significantly higher than the mass contribution reported in previous studies (generally less than 10%) [21,22]. Therefore, the health and environmental impacts of soot particles may be underestimated when based on the mass concentration. It is necessary to conduct in-depth research to further understand their impacts on human health and the environment. Here, we focused on analyzing the particle size distributions and quantitative differences in the soot particles at the urban and rural sites during the different periods (Figure 9).

In terms of the particle size distribution, the soot particles at the urban site had a more concentrated particle size distribution, with steeper waveforms in the simulated functions and equivalent diameters mainly concentrated within the range of 0.1–0.5 μm. In contrast, the particle size distribution was more dispersed at the rural site, with flatter waveforms in the simulated functions and equivalent diameters primarily ranging from 0.1 to 0.8 μm. Based on the distribution of the number of particles, there were more soot particles during U1 than during U2, and the number of particles during R2 exceeded that during R1. Previous studies have shown that there is little difference in the source of single particles in adjacent sampling periods at the same site, and such difference is more likely to be caused by the secondary aging of particles [45,46,47]. Thus, from the perspective of the particle size, the geometric mean diameter was smaller during U1 than during U2, indicating that the degree of aging of the soot particles was higher during U2 than during U1. This may be related to the enhanced long-distance regional transport at the urban site during the Spring Festival (Figure 8b). In contrast to the urban site, the geometric mean diameter was larger during R1 than during R2, indicating that the degree of aging of the soot particles was weaker at the rural site during R2.

### 3.6. Aging Comparison of SIA Particles Coated with OM at the Urban and Rural Sites

SIA particles coated with OM are believed to be formed through the condensation of secondary organic aerosols formed via oxidation of the VOCs on inorganic particles [54]. TEM was utilized to identify whether the SIA particles were coated with OM (Figure 4b) and to directly measure the thickness of the OM coating. During U1 and U2, the SIA particles coated with organic layers accounted for 42.0% and 17.9% of the total observed OM-SIA particles, respectively. However, they only accounted for 25.2% and 8.5% of the total observed OM-SIA particles during R1 and R2, respectively (Figure 6). This indicates that the SIA particles were more prone to be coated with OM at the urban site. Additionally, this process appeared to weaken at both sites during the Spring Festival. 

**Figure 10:**
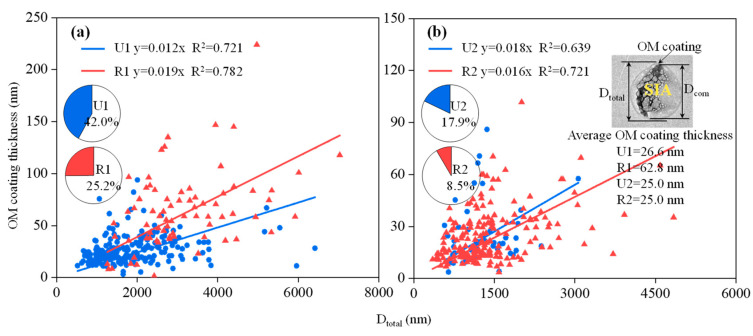
Correlation between the size of the shell and the thickness of the coating for the SIA particles coated with OM during (**a**) U1 and R1, and (**b**) U2 and R2. The pie charts show the number fraction of the SIA particles coated with OM.

As shown in Figure 10, the thickness of the OM coating increased as the diameter of the SIA particles increased, indicating that the SIA particles with thicker OM coatings had a higher degree of aging [29,55]. During U1, the sizes of SIA particles coated with OM were relatively concentrated, mainly within 1000–3000 nm, while during R1, the particle sizes were more dispersed within the range of 1000–6000 nm. This indicates that during the pre-Spring Festival, the finer SIA particles at the urban site were more likely to be coated with OM. In addition, during R1, the thickness of the OM coating was approximately 2.4 times that during U1 (Figure 10b), further indicating that the SIA particles at the rural site had a higher degree of aging during the pre-Spring Festival period. The average thickness of the OM coating was the same (25.0 nm) during U2 and R2. During U2 and R2, the sizes of the SIA particles coated with OM were smaller and more concentrated, primarily ranging from 300 to 2250 nm and from 750 to 1500 nm, respectively. This indicates that the finer SIA particles were more likely to be coated with OM during the Spring Festival, and this process was even more significant at the rural site.

## 4. Conclusions

Despite the extensive in-depth research on atmospheric aerosols in Northwest China, one of the most severely haze-affected regions, there is still a scarcity of information about the mixing state, morphology, and aging mechanism of aerosol particles. In this study, we conducted an observational analysis of atmospheric single particle aerosols in the representative city of Lanzhou and its surrounding areas from the pre-Spring Festival period to the Spring Festival period based on TEM-EDS method. We found that the concentrations of the gaseous pollutants were generally higher at the urban site in Lanzhou than at the rural site. In terms of the different periods, the mass concentrations of most of the air pollutants (i.e., PM_2.5_, CO, and NO_2_) were significantly higher at the urban site than at the rural site before the Spring Festival. However, during the Spring Festival, the mass concentrations of PM_2.5_ and SO_2_ increased at the rural site. Based on their morphologies and elemental compositions, the individual particles were divided into five categories, and the vast majority of the individual particles existed in the form of external mixing. Overall, the urban site predominantly contained OM, SIA, and OM-SIA particles, while the rural site primarily contained OM, SIA, and soot particles. From the pre-Spring Festival to the Spring Festival, the degree of external mixing of the particles increased significantly at both the urban and rural sites, and the proportions of the OM and SIA particles increased the most at the urban and rural sites, respectively. Influenced by the source and degree of aging, significant differences in the particle size distributions were also observed at the two sampling sites. During U1, the air masses primarily originated in the northern and eastern regions of Qinghai, while during U2, they mainly originated in the northeastern part of Qinghai. During R1, the air masses primarily originated in southern Gansu and western Qinghai, and during R2, they mainly originated in southern and central Gansu. The potential source regions of the PM_2.5_ at the urban site were primarily located in central Gansu and eastern Qinghai, while the potential sources regions of the PM_2.5_ at the rural site were located in southern and central Gansu. From the pre-Spring Festival to the Spring Festival, the sizes of the soot particles generally increased at the urban site and decreased at the rural site, highlighting the strong regional transport effect in the urban areas and the reduced aging and mixing degree of the soot particles in the rural areas during the Spring Festival. The finer SIA particles were more likely to be coated with OM, and the finer SIA particles at the urban site had a greater impact on the thickness of the OM coating during the Spring Festival.

## Data Availability

Data will be made available on request (zhangjunke@home.swjtu.edu.cn).
